# Rules and regulations for a pregnant endourologist: the European perspective

**DOI:** 10.1007/s00345-021-03896-y

**Published:** 2021-12-07

**Authors:** Patrick Juliebø-Jones, Amelia Pietropaolo, Anne-Francoise Spinoit, Anne K. Bergesen, Gigja Guðbrandsdottir, Christian Beisland, Nicola von Ostau, Nina N. Harke, Maria J. Ribal, Maria Zerva, Ewa Bres-Niewada, Patricia Zondervan, Liza McLornan, Stefania Ferretti, Ursula Tonnhofer, Ulrike Hendrika Necknig, Andreas Skolarikos, Bhaskar K. Somani

**Affiliations:** 1 Department of Urology, Haukeland University Hospital, Bergen, Norway; 2 Department of Clinical Medicine, University of Bergen, Bergen, Norway; 3 Department of Urology, Ghent University Hospital, Ghent, Belgium; 4 Young Academic Urologists (YAU), Urolithiasis & Endourology Working Party, Arnhem, The Netherlands; 5 Department of Urology, Universitätsklinikum Essen, Essen, Germany; 6 Department of Urology, Hospital Clinic, University of Barcelona, Barcelona, Spain; 7 Department of Urology, Red Cross Hospital, Athens, Greece; 8 Department of Urology, Roefler Memorial Hospital, Pruszkow, Poland; 9 Department of Urology, Amsterdam Medical Centers, Amsterdam, The Netherlands; 10 Department of Urology, Beaumont and Connolly Hospitals, Dublin, Republic of Ireland; 11 Department of Urology, University Hospital of Parma, Parma, Italy; 12 Department of Paediatric Surgery, Medical University of Vienna, Vienna, Austria; 13 Department of Urology, Klinikum Garmisch-Partenkirchen, Garmisch-Partenkirchen, Germany; 14 Department of Urology, National and Kapodistrian University of Athens, Athens, Greece; 15 Department of Urology, University Hospital Southampton, Southampton, UK

**Keywords:** Maternity leave, Radiation, Pregnancy, Endourology, Guideline

## Abstract

**Introduction:**

Working in surgery while pregnant is challenging. Navigating this period safely is of paramount importance. Anecdotal observation suggests that there exists great variation among European nations in regard to maternity leave and radiation safety.

The aim of this article was to gain insight into policy patterns and variations across Europe regarding these issues.

**Methods:**

A series of core question items was distributed to representatives across 12 nations Austria, Belgium, Germany, Greece, Iceland, Italy, Netherlands, Norway, Poland, Republic of Ireland, Spain and the United Kingdom).

**Results:**

The total number of weeks with full pay ranged from as little as 4 weeks in Belgium to 32 and Iceland. All countries included in this study give the option of additional weeks beyond the initial period, however at reduced pay. Some offer unpaid leave beyond this. Only 5/12 countries had a specific policy on when the pregnant surgeon should come off the on-call rota. Only Austria, Italy and Poland stipulate a requirement for the pregnant clinician to be replaced or be completely exempt in cases involving radiation. Only Germany, Iceland, Norway and Poland highlight the need to limit radiation dose in the first trimester. Beyond this, Germany alone provides written guidance for reduction in gown weight and along with Poland, display arguably the most forward-thinking approach to resting.

**Conclusion:**

There is a marked range in maternal leave policies across Europe. There also exists a lack of universal guidance on radiation safety for the pregnant urologist. There is urgent need for this void to be addressed.

## Introduction

A career in surgical specialties is widely recognized to cause difficulties in achieving a good work to life balance [1]. Pregnancy and caring for a newborn child present a further challenge to this equilibrium and it can be difficult to find harmony in navigating this life chapter. Furthermore, working through the course of pregnancy can be arduous both physically and mentally with concerns for the welfare of the mother and the expected child. The recent findings from the United States (US) have revealed that residents take on-average only 8 weeks of paid maternity leave [2]. Moreover, Altierio et al. determined that 42.5% take less than 2 weeks of leave [3]. This research also highlighted that many do not feel supported by fellow residents or their department when taking this time out. Additional obstacles in this regard includes a strain on the program of lack of universal policies. Consideration must also be given to risks for the unborn child and in the setting of urology this includes exposure to ionizing radiation during procedures. However, research by Macdonald et al. highlighted that guidelines regarding procedural safety policies are seldom [4]. Given that the projected workforce in urology is expected to result in more female urologists, this is a subject of great importance and relevance [5, 6]. However, it remains under reported in world literature and anecdotal observation suggests that there exists great variation among European nations in regard to practice patterns and established policies.

The aim of this article was to explore this theme further and gain insight into policy patterns and variations across Europe regarding maternity leave and radiation safety.

## Methods and materials

A series of core questions was delivered to one or more academic endourologists among various European nations affiliated to European Association of Urology (EAU) Young Academic Urology (YAU) groups and EAU section of Uro-Technology (ESUT). This was a fact-based list of questions and not intended to include any subjective elements. One response was recorded from each of the included countries.

The majority of respondents had been through the process of parental leave either as resident and/or consultant and therefore having experience in this area. The questionnaire consisted of two parts: maternity leave and safety policies during pregnancy.

## Results

Information was collected on 12 European countries (Austria, Belgium, Germany, Greece, Iceland, Italy, Netherlands, Norway, Poland, Republic of Ireland, Spain and the United Kingdom).

### Maternity leave

There was considerable range in duration of standard maternity leave for urologists among these countries (14–26 weeks). Total number of weeks with full pay ranged from as little as 4 weeks in Belgium to a maximum of a possible 31 and 32 weeks in Norway and Iceland, respectively. All countries included in this study give the option of additional weeks beyond the initial period, however, the percentage of the previous salary that is paid varies hugely. For example, Poland offers additional leave, which can last up to 32 weeks at 60% of previous wage. In contrast, Greece allows for a further 24 weeks but this is completely unpaid. In certain countries, the mother is prohibited from returning to work after the birth for a fixed period (e.g. Germany—8 weeks).

Several countries offer the clinician to take a pause from their role for a period of time beyond these additional weeks. For example, in Spain and Poland, the mother has the ability to take 3 years of unpaid leave. In this regard, her previous role is protected and the work can be returned to afterwards. Most countries make additional allowances for special scenarios such as sole guardian of the child, multiple birth or if the child is born with a serious illness. Except from Spain where there is no set provision for leaving to commence maternity leave before the expected delivery date, every other country included in this study has a policy to allow pregnant urologists to take up maternity leave earlier. One of the most generous in this aspect is the UK and Norway where the individual can take leave from work 11 and 12 weeks before the due date, respectively. Only 5/12 countries had a specific policy on when the pregnant surgeon should come off the on-call (emergency and out of hours) rota. One of the most flexible system appears to be in Italy, whereby the clinician can relinquish these duties at any time (optional and clinician may decide to continue). In Austria, the rules are stricter and clinicians must come off the on-call rota as soon as the pregnancy is announced to employer. In the other countries, it ranged from week 28 (Norway) to week 32 (Belgium). Further considerations are that in Italy, pregnant urologists do not work night shifts and during the Covid-19 pandemic, they do not have any patient contact during the pregnancy. In Germany, the clinician has the option to relinquish all night shift (or 24 h) duties as soon as the pregnancy is announced. Her salary will remain as it was before.

Variations within a country do exist, for example in Belgium, depending on whether employment is in the private or public sector. For example, weeks with full pay is only 4 weeks in the private sector, but 8 weeks in the public sector (Table 1).

**Table 1:** Maternity leave entitlement

Country	Ordinary maternity leave entitlement (weeks)	Possible additional weeks	Weeks with full pay	Weeks with half (or partial) pay + Statutory maternity pay (SMP)	Weeks with Statutory maternity pay	Weeks with unpaid leave	Week before birth when maternity leave can start	Week to come off on call rota	Extra
Austria	16	121	16	52 (80% full pay)	NS	NS	8	Announcement of pregnancy	
Belgium	15 (public system), 12 (private system)	3–5 if twins or baby not well	8 (public system), 4 (private system)	7 (public system), 0 (private system)	15–20 (public system), 0 (private system)	Any additional time beyond 20 (public system), 12 (private system)	6	32	
Germany	14	148 (44 weeks with 65% partial payment to either parent)	14	44 (with 65% of former net salary either mum or partner with a maximum pay of 1800€/month) Can be extended to 52 weeks in total if partner takes a minimum of 8 weeks too. Also possible 88 weeks, then half amount paid (max. 900€/month)	14 (maternity protection (6 weeks before plus 8 (12 multiple birth) weeks after birth) full net pay	36 months	5	Announcement of pregnancy (relinquished form night duties)	1 year of parental leave (from a total of 3 years) can be possibly taken between second birthday until eighth birthday. In case unborn child is in danger because of work, fully payment until birth plus 8 weeks after birth
Greece	17	24 (unpaid)	17	0	30	0	3	NS	
Iceland	26	6	32 (if additional 6 weeks taken from father’s allocation)	NS	26	NS	8	NS	One year of leave allocated to child – each parent receives 6 months
Italy	21	26	20	26 (30% pay)	30% of total	51	8	No limit	COVID-19 – pregnant urologists do not have patient contact-no night shifts during pregnancy
Netherlands	16	26 (unpaid)	16	0	0	0	6	NS	Additional leave needs to fit schedule
Norway	15 (in addition to 3 obligatory weeks before due date)	15 (paid to either parent)	16 (or 31 if mother gets the additional weeks)	59 weeks for both the parents combined with 80% pay	12 weeks during pregnancy	12 or 24 months if parent has sole responsibility	12	28	Father gets 16 weeks and additional 2 at time of birth
Poland	20	32 (60% pay)	20	32(60%) + 20(100%)	20	36 months	6	NS	
Rep. Ireland	26	16	26	NS	NS	16	4	NS	
Spain	16	2	16	NS	16	Possible till child 3 years	0	NS	
UK	26	26	8	18	13	13	11	32	

*NS *not specified

### Radiation safety and work environment

With regard to the need to change duty if involved in cases involving radiation, Austria, Italy and Poland all stipulate a requirement for the pregnant clinician to be replaced or be completely exempt in these scenarios (Table 2). In contrast, the law in Greece and Belgium does not address this. In the remaining 7 countries, this is optional and observations from respondents were that in reality, the decision is determined locally between the individual and employer. Only Germany, Iceland, Norway and Poland highlight the need to limit radiation dose in the first trimester. Beyond this, Germany alone provides written guidance for reduction in gown weight and along with Poland, display arguably the most forward-thinking approach to resting. For example, the pregnant urologist cannot be in a continuous standing position (e.g. operating) for period of 2 h or more. In addition to this, Germany is the only nation to stipulate a limit on heavy lifting (max. 5 kg).

**Table 2:** Policy on radiation exposure during pregnancy for urologist

Country	Need to change duty (if involved with endourology procedures)	Limit radiation in first trimester	Reduce gown weight	Reduce standing and sitting for long periods	Max dose for unborn child	Lead protection	Additional comments
Austria	Yes	Yes	NS	Times for additional rest	1 mSv	NS	No radiaton duties once pregnancy announced
Belgium	No	No	No	No	1 mSv	0.25–0.35	
Germany	Optional	Yes	Yes	Yes < 2 h Option to rest at any time No shifts working alone allowed	1mSV (monitored weekly)	0.25, 0.35 or 0.5 mm plus 0.35 mm thyroid-gland-protection	Heavy lifting limit 5 kg; no night shifts; max shift 8.5 h; Max 90 h in 14 days
Greece	No	No	No	No	1 mSv	0.25–0.50	
Iceland	Optional	Yes	NS	Required to alternate with colleague and designated rest place	1 mSv	NS	
Italy	Yes	No	No	No	1 mSv	0.25–0.35	
Poland	Yes	Yes	NS	Max 3 h daily	1 mSv	NS	Max 8 h; no night shifts; Overtime not allowed; No heavy lifting
Netherlands	Optional	Optional	No	Optional	No	Standard	No lightweight version
Norway	Optional	Yes	No	No	1 mSv	NS	
Rep. Ireland	No policy	No policy	No policy	No policy	No policy	No policy	
Spain	Optional	No	No	No	1mSV	0.25–0.50	
UK	Optional	No	No	No	1mSV	0.25–0.35	

*NS *not specified

## Discussion

### Key findings

This study provides an overview of maternity leave rights and regulations among European countries. Based on the findings, it seems that as well as great variation, certain countries have more favorable maternity leave allowances with regard to time and financial renumeration. Germany and Poland appear to have the most detailed criteria surrounding radiation safety in the operating room. It is disappointing that many nations do not have clinician specific policies on many of these items including the Republic of Ireland which has none at all. It is therefore left to the individual and their local employer to devise and agree ad hoc plans for these worrying issues. However, a key reason for why there may not appear any obvious clinician specific regulation, e.g. Norway and Iceland, may be because it is covered within more generalized rules, which are embedded in higher order laws at a national level. The latter concern safety in the working environment and therefore radiation exposure for the general population.

### Risk factors for adverse pregnancy outcomes

The importance of this topic cannot be understated. Stress in pregnancy is a known risk factor for adverse outcomes for both mother and unborn child and heavy workload can result in reduced birth weight [7, 8]. Working in an excess of 32 h a week has also been shown to increase the risk of intrauterine growth restriction (IUGR) and reduced birth weight as a result [9]. Fatigue is associated with an increased risk of miscarriage [10]. The European Union (EU) has released a list of possible risks that a pregnant worker can encounter during her daily work [11]. Incorporation of such guidance into user friendly and universal regulations is therefore paramount to support healthy pregnancies.

### Maternity pay

Provision of suitably paid maternity leave support is essential. Aitken et al. conducted a systematic review comparing paid maternity leave versus nonpaid maternity leave [12]. The former provided better maternal and infant health benefits. Stearns et al. demonstrated that since 1978, when paid leave was introduced in the US, the rate of low birth weight and early term birth decreased by 3.2 and 6.6% respectively [13]. In a similar manner, Jou et al., determined that paid maternity leave reduced the odds of maternal and infant re-hospitalization in US [7]. Based on these findings, regulation has changed worldwide and the median number of weeks of paid leave for mothers among Organisation for Economic Cooperation and Development countries, had risen from 14 weeks in 1980 to 42 weeks by 2011 [14].

### Gender variations in workforce

The topic is a matter of heated debate lately as the number of women enrolled in medical school and specialty training programs has increased dramatically over the past several decades [5]. This new wave has led the way for a renewed interest towards parental leave and working mothers needs in the medical field. In 2015, the UK government introduced new flexible leave allowances that encouraged and facilitated for working parents to share leave after the birth or adoption of their child. This allowed both parents in turn, to spend time with their newborn child up to a maximum of 50 weeks’ leave and 39 weeks’ statutory shared parental pay, shared between parents in the first year after birth or adoption. Indeed, other nations now implement a more joint approach. As of 2021, mothers and fathers in Iceland are both allocated 6 months equally. The philosophy behind this approach, which is also shared by other Scandinavian countries appears to be centred around this period of leave belonging to the newborn child and then divided among the parents thereafter. It also highlights the caution needed when interpreting information regarding maternity leave. What appears to be less time in one country may in fact be as a result of that country allocating more time to the other parent.

### Radiation

Radiation protection in pregnancy is based on the knowledge that exposure to 1 mSv increases the risk of congenital malformation by 0.008%, it is, therefore, recommended by International Commission of Radiological Protection (IRCP) that pregnant workers should not be exposed to more than 1 mSv [15]. A standard 0.5 mm lead apron can block up to 99% of radiation and maternal tissues block a further 70% [16]. Uzoigwe et al. concluded that up to 800 orthopaedic procedures can be safely performed before the pregnancy radiation exposure limit is exceeded [17]. However, the latter will vary depending on type of surgery performed. A similar conclusion was drawn by Birnie for radiation exposure among urologists [18]. Comparison between urologist exposure during fluoroscopy and the normal population exposed to environmental radiation, did not show any relevant difference in radiations recorded. Unfortunately, clear and standardised guidance during pregnancy is not always available and often surgeons have to rely on good-will rather than a formal hospital policy. Many pregnant workers are forced to find their own methods to mitigate risk such as by wearing double led gown protection or a lighter 0.25 mm body gown plus a skirt so there is overlap at the abdomen but less weight on the shoulders. Standing two metres away from the image intensifier will also reduce exposure by a factor of four. This can allow the surgeon to safely be in theatre while supervising a junior doctor.

## Further considerations

The perspective on this topic may differ between resident and consultant. For example, the latter may feel more established in their role and more comfortable in highlighting their rights. Although time lost away from training appears to be honored once leave has come to an end, it can be challenging and stressful to spend time away from a craft-based profession, such as surgery. A mother may even therefore prefer the option to share the leave more equally with the father.

Although most countries offer options to for example change duty when performing fluoroscopy, it is arguable that this should be mandatory, which could remove the stress or burden to the pregnant urologist who has to decide this for themselves.

### What is lacking?

As our study confirmed, in many countries the radiation protection policy is clear in regard to maximum radiation exposure. However, the majority have no fixed rule established for lead gown usage, exposure risk during the first trimester and change of duty in case of heavy workload or fluoroscopy usage. Although in some countries such policy is available, often it is only optional and difficult to find, which leaves the female surgeon to feel poorly supported and in a vulnerable position where they feel the obligation to decide for themselves how best to proceed and tackle this taboo subject.

### Limitations

To our knowledge, this is one of the first studies to address the variation in maternity leave and safety regulations for urologists across Europe. Several limitations do exist including that many other European and non-European countries were not represented.

However, we do believe the number collected is sufficient to highlight the diversity in this area and the need for action. Full comparison on all the items included is not possible as many countries appeared to not specify and even have no policy on these matters.

### Recommendations

Development of universal guidance on for example radiation safety in pregnancy by international bodies such as the European Association of Urology (EAU) would be a valuable first step in improving the conditions for pregnant urologists and their wellbeing. These should be easily available and would serve as a blueprint for adoption by the relevant societies of individual nations. While a specific policy for the pregnant urologist may not exist, it may be that is in encompassed by a law on a national level and therefore there does exist protection for the clinician and guidance for the employer accordingly. However, it is our feeling that international and national bodies in urology should address this topic and disseminate transparent guidance to remove uncertainty and worry.

## Conclusion

This study highlights the range of maternal leave policies among European nations and the lack of universal guidance on radiation safety for the pregnant urologist. There is urgent need for this void to be addressed to better support the pregnant urologist and ensure the well-being of them and their unborn child.
